# Integrated review of the nexus between toxic elements in the environment and human health

**DOI:** 10.3934/publichealth.2022052

**Published:** 2022-11-30

**Authors:** Rolf Niede, Dinesh K. Benbi

**Affiliations:** 1 Institute of Geoecology, Technische Universität Braunschweig, Germany; 2 Department of Soil Science, Punjab Agricultural University, Ludhiana, India

**Keywords:** human exposure to PTEs, risk assessment, toxicological effects, therapeutic measures, new treatment options

## Abstract

Emerging pollutants in the environment due to economic development have become a global challenge for environmental and human health management. Potentially toxic elements (PTEs), a major group of pollutants, have been detected in soil, air, water and food crops. Humans are exposed to PTEs through soil ingestion, consumption of water, uptake of food crop products originating from polluted fields, breathing of dust and fumes, and direct contact of the skin with contaminated soil and water. The dose absorbed by humans, the exposure route and the duration (i.e., acute or chronic) determine the toxicity of PTEs. Poisoning by PTEs can lead to excessive damage to health as a consequence of oxidative stress produced by the formation of free radicals and, as a consequence, to various disorders. The toxicity of certain organs includes neurotoxicity, nephrotoxicity, hepatotoxicity, skin toxicity, and cardiovascular toxicity. In the treatment of PTE toxicity, synthetic chelating agents and symptomatic supportive procedures have been conventionally used. In addition, there are new insights concerning natural products which may be a powerful option to treat several adverse consequences. Health policy implications need to include monitoring air, water, soil, food products, and individuals at risk, as well as environmental manipulation of soil, water, and sewage. The overall goal of this review is to present an integrated view of human exposure, risk assessment, clinical effects, as well as therapy, including new treatment options, related to highly toxic PTEs.

## 1. Introduction

Human well-being derives from economic and technological progress but also from the quality of the environment and fundamental life-interests such as health, wealth, expansion of knowledge, and freedom [1],[2]. Economic development, particularly in Europe, North America and China is leading to socio-economic progress, growth in employment and human well-being [3],[4]. However, this development associated with industrialization causes the spread of pollutants, including mutagens and genotoxic carcinogens whose effects may persist in the long term [5]. The growing incidence of a variety of cancers in advanced countries has been drawn back to environmental pollution in particular after World War II [5]–[7]. Humans are exposed to pollutants via food crops, soil, water and air. The spread of the novel coronavirus (COVID-19), inter alia, is due to air pollution driven by industrialization, which is generating higher numbers of COVID-19-infected individuals and deaths worldwide [8],[9].

Potentially toxic elements (PTEs), among several others, are a major group of pollutants with high relevance for human health. Since the emerging metal age, they are extracted from the lithosphere by mining for various economic and industrial purposes. Among the PTEs are “heavy metals” with a density greater than 5 g cm^−3^ which may have a direct or indirect impact on human health. Some of these metals, including Cu (copper), Fe (iron), Co (cobalt), Ni (nickel), Zn (zinc) and Mn (manganese), have functional roles and are essential for biochemical and physiological functions in humans. Other metals including Al (aluminium), Sb (antinomy), Ba (barium), Be (beryllium), Bi (bismuth), Cd (cadmium), Cr (chromium), Ga (gallium), Ge (germanium), Au (gold), In (indium), Pb (lead), Li (lithium), Hg (mercury), Ni (nickel), Pt (platinum), Ag (silver), Sr (strontium), Te (tellurium), Tl (thallium), Sn (tin), Ti (titanium), V (vanadium) and U (uranium) are not essential as they have no established biological functions [10]. However, in high doses some of these metals can be harmful to humans while others such as Cd, Hg, Pb and Cr have deleterious effects even in small quantities causing acute and chronic toxicities in the body. The metalloids Se (selenium) and highly toxic As (arsenic) are also classified as PTEs.

Sources of PTEs are natural and anthropogenic. Natural occurrence is due to the geochemical background in rocks, volcanic eruptions and windblown mineral dust. Concentrations of PTEs in soils are correlated with those of their parent materials [11]. The latter vary widely in PTE concentrations as to the varied mineral composition of different types of rocks. Among anthropogenic sources of PTEs, airborne sources, coal ash, metals from mining, waste disposal, pesticides and (in)organic fertilizers, lead-based paints, petrochemicals and leaded gasoline are the most important [12]–[18]. Urban soils that are under the strong human influence in (sub)urban areas [19]–[21] and mine or quarry soils [22]–[24] are often mixed with PTE-polluted materials, therefore representing hot spots of PTE pollution. Both are classified as Technosols in the World Reference Base for Soil Resources [25], including soils from wastes (landfills, sludge, cinders, mine spoils and ashes), pavements with their underlying unconsolidated materials, soils with geo-membranes and a variety of constructed soils in human-made materials, and roadside soils [26]. Major exposure routes to PTEs include ingestion via direct accidental oral uptake, through indirect ingestion and consumption of polluted food crops [27] and outdoor hand-to-mouth activities mainly by children [28],[29]. The contaminants are also taken up by humans via consumption of drinking water, direct skin contact with polluted water and soil, and inhalation of dust and fumes [15]. Another route of exposure could be through PTE-polluted water flowing via streams and rivers into the sea and thus contaminating fish and sea foods consumed by humans. If exposure to PTE contents exceeds the critical limits, such human exposure may be capable of causing serious health hazards [30],[16]. In the human body, PTEs are known to be persistent exhibiting excretion half-lives of several decades and possibly leading to numerous diseases [31]–[34].

Globally, an estimated 12.6 million fatalities have been reported in recent years caused by more than 100 diseases as a consequence of unhealthy environments including PTE-contaminated soils [35]. Compared to other pollutants, contamination of soils with highly toxic PTEs (e.g., As, Cd, Cr, Hg, Pb) is more harmful to humans [36]. Arsenic, although a metalloid, in numerous studies is regarded as a metal because in many aspects its behavior is similar to that of a heavy metal. In the human body, As is particularly deposited in bones, hair, and other organs. It can cause diseases of the nervous and cardio-pulmonary systems accompanied by cancer [37]. High PTE levels in animals result in reproduction problems, reduced vitality, and the manifestation of mutagenic, teratogenic and cancerous diseases [38].

Risk factors for toxicity of PTEs include the amount and nature of the substance, possible combination of PTEs, route and duration of exposure, nutritional and health status, genetics, age and body weight. Several substances used in complementary medicine may result in PTE toxicity [39]. Symptoms and signs of PTE toxicity can be either due to acute exposure to large amounts, or due to chronic exposure to frequent small quantities which may result in cumulative toxicity. Compared to a single PTE, exposure to multiple PTEs can be more hazardous [40]. Investigations on the PTE status in humans include tests of blood, urine, hair, nail and skin. The top priorities in treatment of PTE poisoning should be preventing any further exposure, removal of the hazardous agent from the body using chelating agents, supportive therapy, and patient education. Public health measures should include prevention or minimizing exposure, monitoring water, air, food, and individuals that are at risk, as well as protection of water, air, sewage and soil against PTE pollution. In this paper, we discuss the most relevant information on human exposure, clinical effects, as well as therapy, including new treatment options, related to highly toxic PTEs, viz. As, Cd, Cr, Hg and Pb.

## 2. Study design

This study represents a narrative review explaining the existing knowledge on the nexus between potentially toxic elements in the environment and human health evaluating the most relevant literature from Pub-Med sources, Scopus, Google and Google Scholar. The sources include mainly scientific journal articles published during the last two decades. The paper integrates comprehensive knowledge of highly relevant PTEs (As, Cd, Cr, Hg, Pb) from human exposure to the management of poisoning.

## 3. Results and discussion

### 3.1. Human exposure to PTEs

**Figure 1.**
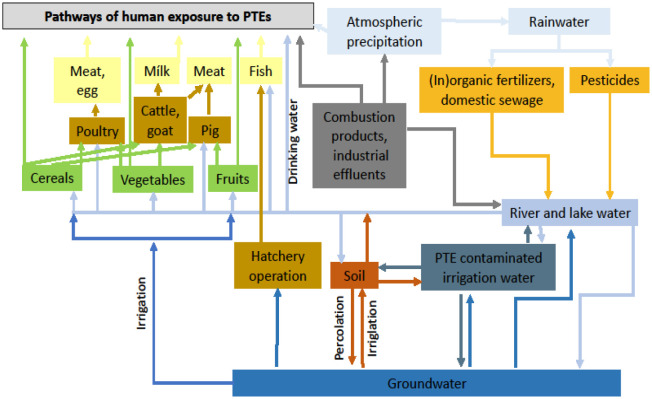
Human exposure to PTEs according to different pathways [adapted from [15]].

The main exposure route by which animals and humans can take up PTEs is through soil and water movement to forage and food plants for consumption. Contaminants can also be ingested directly through drinking water. Polluted water running off to rivers and oceans can contaminate fish and sea foods, which may be eaten by humans. Humans are omnivorous and may thus be endangered through the consumption of PTE-contaminated vegetables, fruits, cereals, meat and fish [15]. Potentially toxic elements may contaminate freshwater bodies such as lakes, rivers and streams and lead to the uptake of PTEs by fish, while in agricultural lands such pollution leads to PTE accumulation in agricultural crop plants. Pollution of the environment with PTEs can also result in contamination of air from sources of natural origin such as volcanic outbursts, windblown dust, steppe and forest fires, marine aerosols, and from various industrial emission sources [15]. People working in specific occupations are exposed to increased risk for particular PTE exposure and toxicity. A schematic diagram representing human exposure to PTEs through different pathways is given in Figure 1.

#### 3.1.1. Arsenic

Arsenic as a ubiquitous non-metallic element can be found in almost all environmental matrices [41]. Arsenic concentration in water is commonly <10 µg L^−1^. However, higher As levels can be found near mining sites or close to mineral deposits [42]. Chronic poisoning with As due to extended exposure to polluted drinking water is a serious issue. In most water sources, As is present as pentavalent arsenate (As^5+^), though in some places the more toxic trivalent arsenite (As^3+^) may occur [43]. An estimated 150 million people globally are affected by As poisoning from polluted water with an increasing trend, as new affected areas are discovered [44]. The largest documented mass poisoning in human history occurred in Bangladesh [45], where between 35 and 77 million people have been affected by severe As contamination. In the Indian state of West Bengal, about 200,000 people have been poisoned with As [15]. After consuming polluted groundwater several million people are at considerable risk of chronic As contamination in Nepal [46] and Vietnam [47].

Human exposure to inorganic As can also occur, although to a lesser extent through consumption of crop plant products and meats. Its concentration in different food products were observed to vary between 20 to 140 ng kg^−1^ [48]. However, if As is present at elevated concentrations in paddy soils, rice may accumulate increased amounts of the pollutant. Under reduced aqueous conditions, As in rice soils exists mainly as mobile As^3+^ which via the silicon channels of the rice plant can be easily transported to shoot and grains [49]. In Bangladesh this has resulted in dietary toxicity after contaminated rice was consumed by humans [50]. The average As intake through food is about 50 µg per day for most individuals [42]. However, children may have additional exposure due to oral uptake of polluted soil material. Natural concentrations of As in soil were found to range from 1 to 40 mg kg^−1^, but disposal of waste and/or application of pesticides can generate much higher As levels [51]. In remote locations, concentrations of As in air commonly are between 1 and 3 ng m^−3^. In cities the levels are much higher and may range from 20 to 100 ng As m^−3^ [42]. Smelter workers and smokers can have exposure to increased concentrations of airborne As trioxide. Inorganic As is poorly absorbed dermally but is strongly absorbed from the gastrointestinal system. To a lesser extent it is taken up through inhalation [43].

In pregnant women, the fetus is at risk of exposure to As contamination, as the contaminant easily permeates the placenta. In mother's body As can also be transferred to the mammary glands and thus to the breast milk [15]. However, even under high exposure As levels in the breast milk were found to be relatively low. Indigenous women in Argentina were exposed to relatively high drinking water As concentrations of about 200 µg L^−1^. However, As concentrations in breast milk were very low and did not exceed 3 µg As L^−1^ [52]. On the other hand, in 1955 in Japan, 100 fatalities were recorded among infants due to polluted powder milk that had As levels between 4 and 7 mg L^−1^ [53].

#### 3.1.2. Cadmium

Cadmium, considered to be one of the most toxic PTEs, can accumulate for up to 2 decades in humans [37]. In Toyama, Japan, the first mass poisoning with Cd in food crops was known after the Second World War when the Jinzu River was polluted with Cd released from mining activities [55]–[57]. The affected people consumed rice having high levels of Cd that was grown in soils irrigated with waters originating from sulfide deposits that were mined for Cu and Zn. Cadmium is also accumulated in the human body via ingestion and inhalation. Smoke of cigarettes is a dominant source of Cd in smoking people. Absorption of Cd in the gastrointestinal tract due to uptake of Cd-containing food is roughly 5% in men and 10% or more in women [58]. Cadmium absorption may be increased by iron deficiency, which commonly causes higher absorption in women [59].

#### 3.1.3. Chromium

Chromium exists in different compounds and can enter the human body via the skin, the gastrointestinal system and through inhalation. Due to excessive exposure Cr can operate as an irritant, an allergen, and/or as a carcinogen in humans [60]. Chromium can pose serious carcinogenic and non-carcinogenic effects in humans when present at concentrations exceeding the tolerable level [36]. Consuming food containing Cr^3+^ is the most frequent route of Cr uptake, as Cr^3+^ occurs naturally in many fruits, vegetables, grains, meat and yeast [15]. Chromium has many industrial applications, such as leather-tanning, pigment industries, chrome-plating, and ferrous and non-ferrous alloy metal production [61]. In a number of epidemiological studies a high lung cancer incidence of workers occupationally exposed to Cr via inhalation has been reported [62].

Hexavalent Cr (Cr^6+^) is much more toxic compared to Cr^3+^. The IARC (International Agency for Research on Cancer) has classified Cr^6+^ as a carcinogen via inhalation as the exposure pathway [63]. An increase of mortality due to stomach cancer was reported in a study in China in an area with elevated Cr^6+^ levels in drinking water [64]. However, up to now there are limited clinical studies on the human health effects of Cr^6+^ following ingestion [15].

#### 3.1.4. Mercury

Routes of human exposure to Hg include air, water, food, soil, and teeth treated with amalgam. Inorganic Hg in the environment is dissolved in water bodies and can be bioaccumulated via the food chain, subsequently taken up by humans and affect their health. Elemental Hg in the body is oxidized to form mercurous and mercuric forms [15]. Poisoning of humans can damage the brain, especially in case of young children [65],[66]. The main route of exposure to elemental Hg is by breathing in volatilized Hg vapor (Hg^0^), followed by dissolution in plasma, blood, and hemoglobin. Under these conditions Hg^0^ can be distributed to body tissues, with high concentrations in brain, CNS and kidneys [15]. In expectant mothers, it can penetrate the placenta and reach the fetus [67]. A risk persists after birth when mother's breast milk is also contaminated. Methylmercury (MeHg) from Hg being present in humans is widely distributed throughout the body and exhibits strongest toxicity. Mercurous and mercuric salts may damage gut lining and kidneys. The first well-documented mass poisoning with MeHg was due to consumption of polluted shellfish and fish in Minamata, a fishing village in Japan, in 1953 [68]. It was considered one of the world's most horrific environmental disasters. The MeHg was emitted to Minamata Bay facing the Yatsushiro Sea from a nearby factory that produced vinyl compounds and acetaldehyde. The clinical picture was officially recognized in 1956 and called “Minamata disease”. A second outbreak of this disease occurred next to the Agano River, in Japanese Niigata Prefecture in 1964 and 1965 [69]. In Kumamoto and Kagoshima Prefectures 2.263 victims of Minamata disease had been documented until the end of November 1999; 1.368 of them died. In Niigata Prefecture, 690 victims of Minamata disease were identified; 338 of them died. Small-scale miners separating gold from ore often use Hg. The hazardous process of extracting Au by Hg blending of ore has the potential of widespread contamination of water, sediments and soil, and of causing adverse health effects in people [70],[71].

#### 3.1.5. Lead

Lead is one of the most abundant heavy metals and its absorption by humans occurs via ingestion of soluble Pb compounds with water, food products and soil or through inhalation of fumes or fine particulates [15]. Toxic effects of Pb cause environmental and human health problems because of its persistence at contaminated sites and its inclusion in complex mechanisms of biological systems. On the basis of 2015 data, the WHO and the Institute for Health Metrics and Evaluation and have reported more than 494,000 deaths and “a loss of 9.3 million disability-adjusted life years” as a consequence of human exposure to Pb [72]. Young children are particularly susceptible to Pb poisoning, since they are strongly affected by ingestion of Pb-contaminated dust or soil. Many children have died from exposure to Pb-polluted soil [72]. The main toxic effects caused by Pb poisoning are neurological. More than 600,000 children worldwide exhibit mental retardation each year as a consequence of elevated blood Pb levels [73]. In children, besides learning and concentration difficulties Pb may cause behavioral disturbances [74].

### 3.2. Risk assessment

The dominant sources of contamination of soil with PTEs include sewage irrigation, mining, sludge application and smelting operations for metallic ores, industrial wastes and combustion of fossil fuels [75]. Highly toxic As, Cd, Cr, Hg, Pb as well as Ni, Cu, and Zn are among PTEs found at contaminated sites worldwide [18]. For risk prevention and pollution control, evaluation of the source-specific human health risk of exposure to PTEs is important. China in particular is experiencing rapid economic and technological advancements and is thus at increased risk of PTEs contamination of soil. Shifaw [75] reviewed more than 100 individual studies to evaluate the PTE pollution status of soils in China. Based on an integrated pollution index, the soils in about 53% of the provinces were moderately to heavily polluted.

In a source-specific study [76] collected a total of 425 topsoil samples (0–20 cm) in the Zhangqiu District of Shandong Province (eastern China), and analyzed concentrations of As, Cd, Cr, Cu, Hg, Ni, Pb, and Zn. Concentrations of all of the PTEs exceeded the background values in soils. Cr and Ni were mainly derived from natural sources, while Cd, Cu, and Zn originated from transportation sources. The average contribution percentages were 24.36% from natural sources, 33.79% from the transportation sector, and 41.85% from industrial sources. In particular the emissions from industry and burning of coal increased soil pollution with Hg, Pb and As. The largest carcinogenic (36.53%) and non-carcinogenic risk (36.01%) for children was attributed to industrial sources, while the largest carcinogenic (34.98%) and non-carcinogenic risk (37.06%) for adults was attributed to transportation sources. These results provide support for effectively preventing PTE health risks in different age groups from source-specific PTE pollution of soils.

Lei et al. [77] analyzed soils polluted with As in a mixed mining and industrial area in southwestern China. Mixed sources, agricultural and mining activities, and As-related smelting activities represented the potential sources of soil PTEs, with contributions of ~30, 26, 23, and 21%, respectively. As-related smelting activities contributed the most to non-carcinogenic risks (adults: 59.0%, children: 57.2%) and carcinogenic risks (adults: 81.8%; children 92.3%), despite the observation that it contributed the least to the accumulation of soil PTEs (21.2%). Non-carcinogenic and carcinogenic risk showed similar trends for children and adults.

In an abandoned Sb smelting site in Anhua county, Hunan province (southern China) the major PTEs in surrounding soils were As, Cu, Ni, Pb, Sb, and Zn [78]. Ecological risk levels of the six PTEs decreased in the order As > Sb > Pb > Zn > Ni > Cu. Non-carcinogenic risk revealed that As, Pb, and Sb posed health risks for children, whilst for carcinogenic risk, the risk values for As and Ni were higher than the limit values for both children and adults. The average contents of Sb and As were 441 and 412 mg kg^−1^, respectively. Anthropogenic sources accounted for more than 70% of Sb, As, and Pb concentrations in soil, indicating a significant proportion of PTEs accumulation. The findings provide a basis for quick determination of the contamination characteristics and risk control of PTEs at Sb smelting sites.

Roadside soils are a prominent example of significant PTE accumulation from vehicles. Guo et al. [26] compiled a dataset of PTEs in roadside soils based on literature published from 2000 to 2021. The evaluation indicated that Cd exhibited moderate pollution and considerable ecological risk. Traffic density and precipitation significantly affected accumulation of PTEs in soil. This study provides valuable information for policy makers to design effective strategies for pollution prevention and risk control.

Food plants are an important exposure route by which humans can take up PTEs. Mohammadpour et al. [79] evaluated the concentrations of PTEs in fruits sold in Iranian markets without tracing the products to their source. The highest mean concentrations (µg kg^−1^) of aluminum (519), chromium (VI) (20.6), iron (803), nickel (60.4), lead (35.5), copper (208), cobalt (6.79), mercury (4.38), zinc (857), manganese (666), arsenic (7.50), and cadmium (4.40) were obtained in black plum, nectarine, black plum, peach, black plum, cherry, pear, cherry, banana, banana, cherry, and cherry, respectively. Total chronic daily intake of PTEs was lower than the tolerable daily intake for consumers. Except for Ni, the cancer risk of individual toxic PTEs was within the safe limit for children and adults, and the cumulative cancer risk of PTEs in some apples and peach was not safe for adults. The intake rate was found to be the most efficient parameter for cancer risk prevention.

Vejvodová et al. [80] analyzed PTE concentrations in soils and market vegetables from vegetable gardens located within the vicinity of unconfined slag deposits from decades of mining and smelting activities in Kutná Hora, Czechia, to evaluate a potential health hazard to communities using these gardens. Total As concentrations in the soils exceeded EU background levels (4.5 mg kg^−1^) by factors ranging between 1.9 and 93. Compared to total PTE concentrations, plant-available concentrations in soils were relatively low. Despite low plant-available PTE concentrations in soils, the concentrations of As, Cd, Pb and Zn in the vegetables (highest concentrations in cucumber, peppers, zucchini) exceeded EU guideline values, indicating a significant health hazard to consumers.

Although PTE concentrations in wastewater effluents are commonly relatively low, yet long-term irrigation with such waters can lead to severe soil contamination, which can pose potential ecological and health risks via PTE accumulation in food plants. Natasha et al. [81] assessed potential ecological and health hazards of As, Cr, Cd, Cu, Ni, Mn, Pb and Zn in wastewater-irrigated arable soils and different crops in selected areas of Vehari, Pakistan. The values of PTEs in soil samples exceeded their respective critical levels by 20% for As, 87% for Cd, 15% for Cu, 2% for Cr, 83% for Mn, 98% for Fe, and 7% for Zn. Some vegetables exhibited a high PTE accumulation index (e.g., 8.1 for onion), indicating severe potential health hazards. To ensure food safety, wastewater irrigation practices need to be managed in respective areas.

Human breast milk may contain pollutants such as metals. According to a meta-analytic study by [82] evaluating 42 of 836 retrieved articles, PTE mean concentrations in mother's milk were 1.84 mg kg^−1^ for Cu, 1.80 mg kg^−1^ for Zn, 1.03 mg kg^−1^ for Fe, 0.60 mg kg^−1^ for Ni, 0.10 mg kg^−1^ for Pb and 0.15 mg kg^−1^ for As and Cd. The highest concentrations of As (2.80 mg kg^−1^), Cd (0.07 mg kg^−1^), and Pb (2.68 mg kg^−1^) were found in the Western Pacific Region (WPRO), the European Region, and WPRO, respectively. The Eastern Mediterranean Region had the highest concentrations of Cu (3.56 mg kg^−1^), Fe (2.78 mg kg^−1^), Ni (3.13 mg kg^−1^), and Zn (5.58 mg kg^−1^). Althouh different risk patterns were observed in various countries, human breast milk was generally safe for infants regarding PTEs. In contrast, high concentrations of some organic pollutants were observed in breast milk in particular in mother's milk of Inuit women in Canada which prompted concerns about possible adverse effects on infant health [15].

### 3.3. Clinical effects

Potentially toxic elements are known to be persistent and bioaccumulative and affect both animal and human health [83]. Dose and duration of exposure decide on a potential development of clinical features including mutagenic, carcinogenic, and/or teratogenic effects. The mechanisms by which PTEs can have a negative influence on different types of tissues, organs, and systems are multiple, and up to now some of these are not completely understood.

Potentially toxic elements are known to affect cell components and organelles including lysosome, mitochondrial, endoplasmatic reticulum nuclei, cell membrane, and enzymes involved in cell division and cellular metabolism, damage repair and detoxification [84]. As levels of PTEs rise in water, soil and air, they may also increase in humans, causing increased risk of chronic disorders, dementia, premature aging, cancer and other diseases. According to several studies, the production of reactive oxygen species (ROS) and physiological stress play an important role in the toxicity and carcinogenicity of PTEs particularly As, Cd, Hg and Pb. The latter toxicants deplete antioxidants in cells, especially enzymes and antioxidants exhibiting the thiol (-SH) group. This can increase the production of ROS such as hydrogen peroxide (H_2_O_2_), superoxide (O_2_^−^) and hydroxyl (HO˙) radicals. Enhanced production of ROS can deactivate the intrinsic antioxidant defense mechanism of cells [85]. Cadmium, Pb, and Hg are known to be nephrotoxic, in particular in the cortical region [86]. Toxicity of Hg strongly depends on speciation of Hg [87]. There is no identifiable threshold of As, Cd, Hg and Pb above which these may affect human health [88]. Consumption of food polluted with As, Cd, Hg, Pb can also strongly decrease levels of Vitamin C, iron and other essential nutrients in the body which can cause reduced immunological defenses, diminished psycho-social faculties, intra uterine growth retardation as well as disabilities due to malnutrition [89]. Many studies exist that demonstrate the link between PTEs and cancer. For example, high levels of Pb in vegetables and fruits in eastern Turkey's Van Province were found to be linked with a high cancer rate of the gastrointestinal tract [90]. In Bangladesh, high concentrations of As in groundwater and some foods have been reported to cause diseases such as (hyper-) keratosis, melanosis, leucomelanosis, gangrene, non-pitting edema and skin cancer [91]. Several studies performed by the WHO have found that worldwide more than 10% of women are vulnerable to infertility as a consequence of their exposure to Pb, Cd, Hg, and other poisons that are known to cause reproductive disorders [92]. Infertility occurs less frequently in men compared to women. Infertility in women is caused mainly by hormonal imbalance as a consequence of endocrine disruption due to poisoning with PTEs which is currently the most frequent cause of female infertility [93]. The main toxicological effects of individual PTEs have been summarized by [15],[88] and [94]–[99].

#### 3.3.1. Arsenic

Arsenic is a well-known carcinogen and considered as one of the most hazardous chemicals worldwide [100]. The majority of cases of human poisoning with As have been linked to exposure to As^3+^ (arsenite) and As^5+^ (arsenate) the first being 2–10 times more toxic than the latter [43]. A dose of 0.01–0.05 g As_2_O_3_ has been found to be toxic and one of 0.3 g is lethal for humans [101]. Arsenic ingested by humans is widely spread in the body and reduced to As^3+^ which is subsequently methylated to MMA (monomethylarsonic acid) and DMA (dimethylarsinic acid). It is also possible that MMA and DMA are taken up directly which can result from uptake of the pesticides monosodium methylarsenate and cacodylic acid. Both of the methylated forms are removed from the body in particular with urine [102]. Elimination half-lives of inorganic As and its metabolites range between 2 and 4 days [103].

Toxicity of As mostly is due to the ability of its inorganic forms to substitute phosphorous in various biochemical reactions and to interact with the R–SH functional group (also named thiol or sulfhydryl group) of proteins and enzymes. More than 200 enzymes can be inhibited in the human body through interaction of As^3+^ with R-SH groups of proteins [42]. Other effects of reactions with As^3+^ include alteration of gene expression and interference in signal transduction pathways [15]. Arsenate (As^5+^) can replace phosphate, the latter being involved in many biochemical reactions and pathways [104]. These reactions can decrease energy production and enhance oxidative stress, endothelial injury and cytotoxicity [105]. The presence of As^3+^ in the body inhibits the enzyme succinate dehydrogenase which causes reduction of ATP (adenosine triphosphate) generation. By binding to the R-SH groups of dihydrolipoamide As^3+^ also inhibits pyruvate dehydrogenase [15]. Cell uptake of glucose, gluconeogenesis, fatty acids oxidation, and glutathione production may be also impaired.

There are several skin diseases, such as hyperpigmentation, hyperkeratosis, and several forms of skin cancers which are promoted by chronic exposure to As. The most common skin change due to prolonged As exposure is hyperpigmentation. Bowen's disease, which is a form of early skin cancer, is another effect of As exposure. Arsenic hyperkeratosis is widespread and commonly affects the palms and soles, but it can also spread over the arms, dorsum of the hands, fingers, legs and toes. Several Bowen's disease and hyperkeratotic lesions generally can develop invasive malignancies [106].

Symptoms as a consequence of exposure to large amounts of As^3+^ include hemorrhagic gastritis with nausea, vomiting and diarrhea, which can lead to dehydration and shock. Renal failure, hepatotoxicity, peripheral neuropathy and cardiac arrhythmias may also occur with high As levels or after survival of an acute As overdose [15]. At less than acutely toxic doses, peripheral vascular disease, sensorimotor neuropathies, portal hypertension (noncirrhotic) and hematocytopenias may be caused following chronic intake of As, e.g., by long-term uptake of drinking water with elevated As levels [102],[43]. These effects may need several years for development. Chronic elevated As intakes have also been linked to neurodevelopmental effects in children, hypertension and diabetes [107],[43]. The CNS suffers from cognitive impairment due to consumption of As. Poisoning with As also causes changes in neurotransmitter balance and synaptic transmission [108]. Long-term exposure to As has been considered as a cause of lung, bladder and skin cancer [102]. The risk of cancer is promoted by attaching of As to DNA-(deoxyribonucleic acid-) binding proteins and negatively influencing the process of DNA repair [109].

Inorganic As in males impairs reproduction by weight reduction of the testes and reducing the sperm number in the epididymis. Moreover, inorganic As exposure can also affect testosterone and gonadotropin production and disturb the steroidogenesis process [110]. Arsenic consumption in females is associated with an enhanced endometrial cancer risk [111]. Exposure to As during pregnancy may promote endometrial angiogenesis, the latter being critical for embryo development. Clinical signs of endometriosis, subfertility, prematurity, sterility, and miscarriage may be a consequence of these conditions [112].

According to several studies significant variability between individuals in the accessibility to As poisoning exists. Genetic factors were found to be the primary source of this variability [109]. Genotoxicity due to As exposure results in alteration of DNA, which includes chromosome abnormalities, deletion, sister chromatid exchange, and production of mutation micronuclei [113]. Several investigations have been performed to investigate the mechanism of the genotoxic impact of As, including the induction of oxidative stress and the disruption of the process of DNA repair [114]. Arsenic is considered as a weak mutagen and has been demonstrated to have not a direct influence on DNA. However, despite its relatively low mutagenicity, it impacts the mutagenicity of other carcinogens. For example, an increased mutagenicity of As in human cells has been found when they were exposed to UV light [115].

#### 3.3.2. Cadmium

Cadmium is very toxic, almost as much as Pb and Hg [67]. Exposure to Cd can cause damage to different organs, including liver, kidneys, testicles, lung, CNS (central nervous system including brain), bone, and blood system together with cancer. The serious illness caused by Cd poisoning after World War II in Toyama, Japan is known as “itai-itai”, a syndrome expressed by weakening of the joints and bones [55],[56]. The term “itai-itai” was created by the local population for the severe pain with movement associated with the disease. Kidney dysfunction was identified as the cause of osteoporosis. The disease was also observed in other regions of Japan with elevated Cd levels in food crops and its association with renal damage and bone disorders have been confirmed epidemiologically [57]. In patients having “itai-itai” disease mortality risks were increased substantially. Diseases of kidneys and urinary tract were the main causes of death in patients exhibiting renal tubular dysfunction. When Cd-containing compounds are inhaled the first symptoms include headache, cough and fever [101]. Low to moderate exposure to Cd may cause diabetes [116], carotid atherosclerosis [117], peripheral vascular disease [118], chronic kidney failure [119], heart attack [120], hypertension [121], heart failure and stroke [122]. In the general population of the USA, cadmium was associated with an increased risk of cardiac death [123].

Neurodegenerative defects like Parkinson's disease, Alzheimer's disease, multiple sclerosis and amyotrophic lateral sclerosis can result from neurotoxicity caused by Cd [124]. Cadmium markedly affects the functionalities of CNS and PNS (peripheral nervous system) [109],[125]. Clinical manifestations of exposure to Cd include neurological disturbances, olfactory dysfunctions, peripheral neuropathy, mental retardation, learning disabilities together with behavioral changes and the impairment of motor function in children and adults [109]. Additionally, several categories of cell activity including proliferation, cell differentiation and cell death are impaired. The cause of Cd neurotoxicity is death of neural cells via apoptosis which provides apoptosis-induction factors, such as endocrine disruption, impairment of neurogenesis, offering epigenetic effect, inhibition of neuron gene expression, and others [126].

Chronic exposure to Cd causes its accumulation in kidneys and liver where it is attached to metallothionein, a metal-binding protein. A major part of Cd in the body is bound in the kidneys [127], with a half-life ranging between one and four decades [59]. Long-term exposure to high Cd doses can result in glomerular and renal tubular damage, accompanied by decreased glomerular filtration rate and irreversible proteinuria [128]. Increased levels of Cd in urine or blood and the occurrence of different biomarkers of renal tubular effects may result from low level environmental exposure to Cd [129],[130]. Cadmium, accumulating in the liver, is associated with various hepatic dysfunctions. Oxidative stress and hepatocellular damage are a result of Cd-induced changes in the cellular redox balance [131]. Cadmium-induced hepatotoxicity causes liver failure and can promote the risk of cancer [132]. Environmental and occupational exposure to Cd can cause immunosuppressive effects. At low exposure to Cd, humoral immune responses are increased, whereas at higher Cd exposures the effects are not yet completely understood [109].

In an area of China, severe environmental pollution with Cd was associated with highly elevated levels of Cd in the urine of local residents [133]. Increased calcium and phosphorus excretion and decreased hydroxylation of metabolites of vitamin D was the result of advanced renal tubular damage. Postmenopausal women and older adults exhibiting increased urine Cd levels as a consequence of decreased bone mineral density have an increased osteoporotic fracture risk [129].

Severe exposure to fumes and airborne dusts can result in severe pneumonitis [134]. Chronic exposure to particulates containing Cd was associated with worsening of the pulmonary function and emphysema [135]. Decreases in olfactory function were shown to be a result of chronic workplace exposure to airborne Cd particulates [136]. An inverse relationship was documented between Cd in maternal blood, cord blood and birth weight [137]. Cancer in humans has also been associated with Cd. For example, various studies conducted in workplaces have shown a significant increase of mortality due to lung cancer caused by chronic Cd inhalation [138].

#### 3.3.3. Chromium

The toxicity of Cr varies significantly, depending on its chemical form (ion, oxide, hydroxide, (nano-)particle, valence ranging between 2 and 6) [67]. The principal cause of toxic responses is the hexavalent Cr ion (Cr^6+^), whereas trivalent Cr- (Cr^3+^-) containing materials have been regarded as irritants, but not as allergens or carcinogens. A number of severe dermatological effects (acute and chronic) are caused by dermal exposure to substances containing Cr [139]. The powerful oxidizing properties and strong acidity of Cr ions are considered the main causes of its irritant effect on the skin. Systemic contact dermatitis, contact dermatitis, and cancer of the skin are a result of dermal exposure to Cr. Systemic contact dermatitis is triggered by systemic contact with an allergen, which causes the skin to become sensitive through direct dermal contact, while contact dermatitis shows delayed hypersensitivity as a result of periodic dermal exposure to allergens [109]

Concentrations of Cr in the urine were increased following human exposure to Cr^3+^ by inhalation which indicates respiratory Cr absorption. Inhalation of Cr^6+^ compounds induces strong irritation of the respiratory system [60]. Exposure to Cr concentrations in the air >2 µg Cr m^−3^ during a couple of hours may cause rhinorrhea, perforation of the nasal septum, ulceration, pneumoconiosis, asthma and bronchitis [101].

Oral human uptake of CrCl_3_ (chromic chloride) resulted in poor absorption of Cr^3+^ following oral ingestion. Only about 0.5% of the dose was excreted in the urine, but 99% was recovered in the feces [15]. Hexavalent Cr in the blood is absorbed selectively by erythrocytes, reduced to trivalent Cr, and bound to hemoglobin, while Cr^3+^ combines with plasma proteins like transferrin [140]. As a consequence of complete reduction of Cr^6+^ during transport in the blood, only Cr^3+^ was found in the urine [141]. For adults, the lethal oral dose was found to be 50–70 mg soluble Cr per kilogram of body weight [60]. Clinical symptoms of acute Cr intoxication include diarrhoea, vomiting, hemorrhage with blood loss into the digestive system, and cardiogenic shock [142]. The main effects following oral uptake of toxic Cr doses are necrosis of kidney and liver if the patient survives for more than about one week [139]. Long-term exposure to Cr^6+^ was reported to cause failure of liver and kidneys, hemolysis and anemia. Exposure to Cr was also found to induce allergic reactions and hypersensitivity [60].

Chromium has a negative impact on the immune system of humans. High concentrations of Cr^6+^ reduce the humoral immune response and the phagocytic activity of alveolar macrophages [143]. Workers of the mining and consuming sectors that are in contact with Cr have shown to be at increased risk for cancer [144]. Experiments with living and cultured cells have demonstrated that Cr^6+^ causes damages to genetic material, including nucleotide strand breakage (intrastrand and interstrand cross-links of DNA) [145].

#### 3.3.4. Mercury

Mercury is strongly toxic to all living organisms, including humans, and can bioaccumulate, especially in children [67]. Inorganic Hg, which is mostly taken up via ingestion, can be irritating in the gastrointestinal tract [146]. The poisonous effect and toxicity of MeHg (methylmercury) can vary depending on its form, individual susceptibility, absorbed dose, and inflow path [147]. In the body, MeHg reacts with glutathione to form MeHg-glutathione which is distributed via the bloodstream to different tissues and organs. Growing hair, a useful marker of exposure, accumulates MeHg [148]. About 90% of MeHg is distributed in erythrocytes and proportional to the blood concentration the MeHg concentration in organisms is relatively stable [147]. Initially, Hg concentrations in blood decline quickly with a halflife of about 1–3 days, with a subsequent half-life of several weeks [149]. Mercury is mostly excreted by fecal but also renal pathways. Maximum levels in the urine can lag behind those in blood by a few days up to a couple of weeks [149]. Maximum 15% of inorganic Hg is absorbed during its passage through the gastrointestinal system [150]. In contrast, the portion of MeHg absorbed from the digestive tract may amount up to 90% [101]. In human tissues MeHg is dealkylated to inorganic Hg [6].

Methylmercury can be accumulated in the brain of fetuses after the passage through the placenta and the blood-brain barrier. It is very problematic that the concentration of MeHg in the cord blood is about two times higher compared to the blood MeHg level of the mother [147]. This difference is because compared to the mother, fetuses have higher levels of hemoglobin. As the MeHg is transported through the human body combined with glutathione and hemoglobin, and the half-life of MeHg is long, it is present in fetuses and in infants for a long time. Excessive prenatal Hg exposure can result in cerebellar ataxia and palsy, mental retardation, altered physical growth, dysarthria, limb deformities and sensory impairments [151]. Another serious problem is that MeHg and inorganic Hg accumulates in human breast milk [152].

Several skin infections including pink disease (acrodynia) are caused by compounds that contain Hg. Acrodynia is a typical dermatological ailment. When the skin is exposed to metals, especially Hg, it changes the color to pink [153]. People whose skin has been tattooed with the red pigments Cd sulfide and Hg sulfide may be affected by inflammation which is restricted to specific skin areas commonly within about half a year after tattooing [154]. Clinical signs of acute contact dermatitis caused by substances containing Hg include skin irritation, moderate swelling, vesiculation and scaling [109].

Elemental Hg vapor in high doses can cause severe pneumonitis. At chronic Hg^0^ doses below those leading to acute injury of the lung, clinical signs may include irritability, tremor, gingivitis, behavioral disturbances, neurocognitive disorders, anorexia, short-term memory loss, fatigue, sleep disturbance and depression [155],[156]. As MeHg affects the CNS, symptoms particularly include paresthesias, dysarthria, ataxia, hearing loss and progressive constriction of the visual fields [15]. Mercury accumulates in the kidneys where it can cause necrosis of the tubular epithelial cells. Moreover, Hg interacts negatively with dopamine, an essential hormone, and thus has an endocrine effect [157]. There also exists a correlation between MeHg exposure and cardiovascular toxicity [147],[158]. Occurrence of Hg in hair has been linked to acute coronary failure, atherosclerosis and Ox-LDL (oxidized low density lipoprotein) levels in atherosclerotic lesions [159]. Likewise, Hg deactivates paraoxonase which is an antioxidative extracellular enzyme associated with HDL (high density lipoproteins) dysfunction [160]. This mechanism is directly linked to an elevated risk of cardiovascular disease, acute myocardial infarction, coronary heart disease, carotid artery stenosis, and the progression of atherosclerosis [161].

The peroxidative activity of Hg produces ROS that can promote protumorigenic signaling and growth of cancer cells. The contribution of ROS to carcinogenesis is by damaging DNA, cellular proteins and lipids, which results in cell damage [162].

#### 3.3.5. Lead

Following uptake, Pb in the blood is bound to erythrocytes and is subsequently transported to bones and tissues. Bones in particular act as a storage system for Pb [15]. Correspondingly, in Pb-exposed adults, up to 70% of Pb in blood may originate from the bone system [163]. In pregnant women, Pb can cross the placenta and reach the brain of fetuses. It may also enter the babie's body via breast milk of lactating women. A correlation exists between levels of Pb in mother's milk and in the blood of corresponding infants [164]. Lead is commonly removed from soft tissues and the blood with up to 2 months half-life. From bone tissue, Pb is lost considerably slower, with an elimination half-life of several years to decades. The major part of Pb output (about 70%) occurs via urinary excretion, lesser elimination takes place via the feces, and significantly smaller Pb amounts are eliminated via hair, nails and sweat [165].

As lead interferes with the physiologic functions of Fe^2+^, Ca^2+^ and Zn^2+^, the toxic effects result from inhibiting enzymes. Human exposure to Pb inhibits δ-ALA-D (δ-aminolevulinic acid dehydrase) which is an enzyme that catalyzes the transformation of δ-ALA (δ-aminolevulinic acid) to porphobilinogen [101]. The levels of δ-ALA, a unique diagnostic parameter, then increase. Other mechanisms of Pb poisoning include changes in gene expression [166]. Several diseases caused by Pb poisoning are sub-clinical while others exhibit clinical symptoms. Lead intoxication exerts serious health consequences on all organs, but its major impact is on the kidneys [109]. Chronic renal disease caused by high levels of Pb can be characterized by renal failure, interstitial fibrosis, hyperplasia, atrophy of the tubules as well as glomerulonephritis. Lower level exposure to Pb may cause small decrements in kidney function [167]. Chronic Pb exposure has also the potential to damage the liver. Poisoning with Pb can result in depletion of glycogen and cellular infiltration, finally causing chronic cirrhosis [168]. Exposure to Pb can also affect the immune system and may cause a number of immune responses, such as infectious diseases, increased allergies, autoimmunity, as well as cancer [169]. By releasing ROS, Pb is a carcinogenic substance which damages the DNA repair mechanism, chromosomal structure and sequence, and cellular tumor regulating genes. By shifting zinc from certain regulatory proteins, Pb disrupts gene transcription [170]. Poisoning with Pb is associated with a high risk of cancers of stomach, bladder and lung [171]. Movement disorders and anomalous perception during growth and development of the fetus are also consequences of Pb exposure.

Lead present in the bone tissue is mobilized continuously into the blood stream which may be responsible of mental deficiency in humans. In infants and children this is more common compared to adults, as the barrier between the blood and the brain of the young human beings is less effective [172]. This demonstrates that the brain of this group is most vulnerable to Pb poisoning. However, adults as well may be affected by brain dysfunction due to chronic Pb exposure [173]. A large proportion of children from mothers with chronic Pb exposure exhibit symptoms of anemia and mental retardation, and suffer seizures [172], [174]. Dental caries may also be a result of prenatal and postnatal Pb exposure [175]. It has been reported that neurodevelopmental disorders in children may occur at Pb concentrations <10 µg Pb dL^−1^ [176], whereas in adults, neurocognitive dysfunctions have been reported at blood Pb levels between 20 and 30 µg Pb dL^−1^ [177]. Seizures, peripheral neuropathy and overt encephalopathy were observed at Pb concentrations greater than 200–300 µg Pb dL^−1^.

Exposure to Pb for a long time may also cause atherosclerosis, thrombosis, and cardiac disease [109]. Chronic Pb exposure also increases arterial pressure [178]. In pregnant women, low Pb levels were linked with hypertension, premature birth, as well as spontaneous abortion [179],[180]. In men, blood Pb concentrations >40 µg Pb dL^−1^ from chronic high dose occupational Pb exposure resulted in in decreased fertility through altering sperm morphology, and reducing sperm count [181].

### 3.4. Therapy

For confirming PTE toxicity, the examination of blood, urine, hair, nail, and tissues is a common practice. With early detection, the prognosis is commonly not bad. Serious toxicity combined with delayed diagnosis can result in bad prognosis. The level of PTEs in the blood or urine will not accurately reflect body stores from chronic exposure as it reflects solely current exposure. In patients with high body stores, symptoms may not correlate with PTE blood levels, and profound symptoms may be apparent in individuals with blood levels only slightly above normal. In some cases a chelation challenge is necessary to strengthen the diagnosis. Ancillary tests like hemogram to detect anemia, renal and liver function tests are often helpful.

As the first therapeutic measure the patient is to be removed from exposure to PTEs. Once in the body, the hazardous substance may be removed using activated charcoal and by gastric lavage. Skin decontamination is recommended following dermal contact with PTEs [40]. Supportive care is to be applied in the form of intravenous fluids, ventilatory, circulatory and oxygen, as required. In severe cases, extracorporeal membrane oxygenation, hemodialysis and plasma exchanges may be essential.

#### 3.4.1. Administration of synthetic chelating molecules

Specific therapy to remove PTEs from the bloodstream conventionally is by the administration of synthetic chelators. The process of chelating (derived from “chelos”, the Greek word for claw), comprises the fixation of an ion (usually a cation) within a ring structure by a chelating organic molecule. When used together with antioxidants, chelators may be more effective [182]. Electron-donors on the chelators commonly include oxygen, nitrogen, and/or sulphur atoms. Chelators are able to mobilize PTEs from tissues and transporting the chelate moiety to the kidneys to excrete the pollutant through the urine, and to the liver for removal via the bile [183]. The properties of an ideal chelating agent are to form non-toxic compounds, the ability to bind most PTEs, high solubility and cell permeability, and a high elimination rate. However, up to now no such ideal agent exists, and further research is thus urgently needed. Due to several drawbacks of existing chelators, investigations have also focussed on naturally occurring phytochelatines. These may offer a safer and cheaper alternative, especially in developing countries where the problem of poisoning with PTEs is manifold [184].

In case of poisonings with As, chelators like 2,3-dimercaptopropanol (British Anti-Lewisite: BAL) and the derivates D,L-2,3-dimercapto-1-propanesulfonic acid (DMPS) and meso-2,3-dimercaptosuccinic acid (DMSA) are generally suitable antidotes for treatment of humans [101]. However, DMSA and DMPS are more soluble in water than the BAL, and have been shown to be more effective [40]. DMPS together with As creates a stable, five-membered ring complex which can be effectively excreted via the urine. In As toxicity, the benefits of chelation exceed the side-effects and prevents acute renal failure. Laboratory animal experiments have demonstrated that application of DMPS combined with colestyramine cuts off the entero-hepatic cycling of the As-DMPS compound which increases As excretion in the feces.

The treatment of acute Cd intoxication due to oral uptake or inhalation of Cd-containing substances is mostly symptomatic [101]. Fresh and pure air should be provided immediately and airways should be kept free after inhalation of Cd vapors. Following a latency period of a couple of hours, edema of the lungs may develop, causing damage to the pneumocytes. Uptake of high doses of glucocorticoid via the respiration tract is indicated as a first aid. In case of acute poisoning by inhalation, treatment with BAL is recommended. Exposure to Cd via ingestion demands gastric lavage or induction of vomiting. The use of activated charcoal is also useful. In case of chronic Cd intoxication via inhalation or ingestion, BAL should not be applied because Cd may be redistributed from the tissue to the kidneys and may thus damage the latter [15]. Alternatively, in chronic Cd intoxication treatment with a combination of deferiprone and deferasirox can be of great help [185].

In case of Cr ingestion, vomiting should be induced and subsequently a lot of water is recommended to be consumed. For transforming Cr^6+^ into less toxic Cr^3+^, the uptake of ascorbic acid is helpful. In case of Cr poisoning DMPS is an effective antidote. After skin contact with Cr containing substances, the affected areas of the skin should be immediately rinsed with plenty of water. Application of the chelator calcium disodium ethylenediaminetetraacetatic acid (CaNa_2_-EDTA) dissolved in polyglycol is also recommended [15].

Treatment with BAL as an antidote is recommended for intoxication with inorganic Hg compounds. However, BAL should not be applied in case of poisoning with Hg^0^ and organic mercury compounds because the Hg accumulates in the CNS [101]. For intoxication with Hg^0^, Hg^+^ and Hg^2+^, both DMPS and DMSA have proved to be effective antidotes. Additional consumption of vitamin E and selenium can be helpful in the treatment of Hg toxicity [66].

Treatment of Pb poisoning includes chelation therapy particularly in presence of noticeably elevated blood Pb levels and/or severe symptoms. Effective antidotes in humans include CaNa_2_-EDTA, D-penicillamine, DMSA and BAL [101]. However, DMSA has been found to be superior to BAL for treatment of Pb poisoning [40].

#### 3.4.2. Treatment with natural products

There are a number of treatment methods which have been applied to counteract PTE toxicity. Several studies have been undertaken to appraise potential treatment options of substances other than synthetic chelators. These include various bioactive natural products. Natural molecules were presented to be efficient in the treatment of adverse consequences of PTE poisoning [42],[186]. In a number of laboratory animal experiments using various medicinal herbs and other natural products, artificially induced Hg toxicity was significantly reduced [187]. The major advantages of natural product-based medicines besides their noticed efficiency include low incidence of hazardous adverse effects and relatively low costs [188],[189]. Literature surveys reveal that up to now only experimental studies have been conducted in pursuit of medicinal plants and their constituents, i.e., phytochemicals that were shown to reduce PTE toxicity in test animals. In a recent review by [109] natural bioactive molecules potentially suitable for treatment of PTE poisoning were summarized and grouped according to clinical features (Table 1).

**Table 1.** Options for treatment of PTE poisoning with natural products according to clinical features [adapted from [109]].

Clinical feature	Natural product
Neurotoxicity	Para-amino salicylic acid, curcumin, gallic acid, protocatechuic acid, melatonin, apigenin, omega-3 fatty acid
Nephrotoxicity	Curcumin, quercetin, royal jelly, protocatechuic acid, rosmarinic acid, thymoquinone, alpha lipoic acid, wogonin, silymarin, dimercaptosuccinic acid, piperine, gamma-glutamyl cysteine, simvastatin, eugenol, silymarin
Carcinogenicity	Monoisoamyl dimercaptosuccinic acid, andrographolide, curcumin, sulforaphane, melatonin, piperine, quercetin, metformin
Hepatotoxicity	Salidroside, berberine, carnosine, thymoquinone, curcumin
Cardiovascular toxicity	Vitamin C, sulforaphene, curcumin
Skin toxicity	Psoralen, epigallocatechin-3-gallate
Immunological toxicity	Pterostilbene, myo-inositol, curcumin
Reproductive and developmental toxicity	Lutein, ellagic acid, ferulic acid
Geontoxicity	Epigallocatechin gallate, curcumin

These findings (Table 1) were mainly demonstrated in lower animal models. However, they may have significant potentials with respect to reduction of PTE toxicity and to preventive mitigation in people susceptible to environmental exposure to PTEs. These apparently preliminary investigations could serve as stimulation for further studies which can promote discovery of useful agents other than synthetic chelators in clinical treatment of PTE toxicity. The options shown appear to be motivating for further promising pre-clinical and well-designed clinical studies on natural product-based medicines for PTE detoxification therapy.

## 4. Conclusions

Potentially toxic elements are known to be highly hazardous and thus adversely influence human health. Each year a large number of people die as a result of exposure to both naturally and anthropogenically occurring PTEs. Excessive PTE levels cause significant damage to every organ of the body and can display neurological defects, respiratory disorders, gastro-intestinal obstruction, osteoporosis, carcinogenicity, and many others. Compared to adults, young children are more vulnerable to PTE toxicity. A previously active and healthy child may present with distractibility, poor play, anemia, abdominal pain, falling grades at school, and retarded development. Inhalation of PTEs leads to respiratory symptoms, topical contamination, skin lesions, while ingestion causes acute gastroenteritis or dysentery. While acute PTE exposure is commonly dramatic in presentation, chronic exposure is difficult to detect and needs a high degree of suspicion. Exposure to combinations of PTEs will cause more profound symptoms than one element alone. As the knowledge is still limited in this context, further research is required for better understanding the complex molecular mechanisms as well as the human health consequences of exposure to more than one PTE.

Symptomatic treatments and application of synthetic chelating agents have been conventionally used in counteracting PTE toxicity combined with avoiding occupational or environmental exposure. However, these conventional agents are not devoid of side-effects. For example, some metal ions are redistributed to other tissues like the brain and thereby increase neurotoxicity. Synthetic chelators may also deactivate essential trace elements and produce a state of deficiency. Natural products may be a powerful alternative to treat the adverse consequences. However, the current research is limited to laboratory animal experiments and studies on clinical investigations up to now are not available in the literature. Systematic research in this area could help to develop effective natural products for clinical management of PTE toxicity in humans.

From the social point of view, preventive strategies need to be implemented. To lessen the toxic effect of PTEs, potential victims should first be removed from the source of exposure. In addition, it should be aimed at reduced use of PTEs in industrial processes. Workers should not get in touch with chemical products containing PTEs. People with occupational exposure to PTEs and others at risk should receive proper timely and periodic screening for toxicity from PTEs. They should take adequate precautions to prevent or minimize exposure and cooperate with the health authorities. Particular care should be taken with the protection of young children and at-risk individuals from being poisoned. Health education, in this regard, should be reinforced periodically.

The dominant sources of PTEs in the environment include sludge application, mining and smelting operations for metallic ores, industrial wastes and combustion of fossil fuels. The first steps to combatting pollution with PTEs include locating and controlling of these pollution sources. In recent years, progress has been achieved with respect to identification and evaluation of the source-specific health risk of exposure to PTEs, particularly to polluted soils. However, when comparing peer-reviewed studies related to PTE contaminated soils, some limitations become obvious. For example, the number of soil samples per unit area across different study locations is commonly not uniform. Moreover, sample distribution over investigated areas is generally not spatially uniform and may therefore not represent the overall soil contamination situation.

In many polluted areas, humans are exposed to combinations of PTEs, commonly with different health risk from individual PTEs. In contaminated areas, remedial actions need to be been taken to minimize PTE levels in soil and/or to limit their mobility into the food chain. Technologies mostly used for remediation of PTE polluted soils include engineering techniques (soil replacement, soil washing, thermal desorption, electrochemical remediation, solidification, vitrification) and phytoremediation, the latter including the categories phytoextraction (removal of PTEs by metal accumulating plants), phytovolatilization (evaporation of pollutants (e.g., Hg) from aerial parts of the plant) and phytostabilization (based on plant roots' ability to reduce PTE mobility). The major disadvantages of engineering techniqes are that they are expensive, energy intensive, and disturb the soil (in particular soil structure and organic compounds). Sometimes, polluted soil treated with engineering techniques remains useless as a medium for plant growth. By contrast, phytoremediation for soil remediation has been reported to be non-intrusive, effective, inexpensive, socially accepted and aesthetically pleasing.

In regions where such remediation technologies are no available, other approaches may be helpful to reduce the PTE transfer from soil to plants. Application of organic (e.g., crop residues, manures, compost, bio-solids) or inorganic (e.g., limes, phosphates, minerals, industrial co-products) amendments may help to effectively reduce the phytoavailability of different PTEs in soils. Crop selection is effective to reduce the PTE transfer into the human food chain, since different food crops vary in the potential of taking up toxic elements. For example, the uptake of PTEs by peas, beans, tomato, melon and pepper is low, while it is high for spinach, mangold, lettuce, endive and carrot. The latter species should not be cultivated on contaminated soils. If soils are extremely contaminated, cultivation of industrial plants (non-food fibre plants and energy crops) has been considered as a remediation option. However, in world regions with rapid industrialization, urbanization and agricultural intensification, effective control of contamination sources and remediation of polluted soils will remain complicated.

## References

[1] Coccia M, Bellitto M (2018). Human progress and its socioeconomic effects in society. JEST.

[2] Coccia M, Farazmand A. (2019). Comparative Institutional Changes. Global Encyclopedia of Public Administration, Public Policy, and Governance.

[3] Coccia M (2018). An introduction to the theories of national and regional economic development. TER.

[4] Coccia M (2021). Effects of human progress driven by technological change on physical and mental health. Studi Sociol.

[5] Coccia M (2015). The Nexus between technological performances of countries and incidence of cancers in society. Technol Soc.

[6] Irigaray P, Newby JA, Clapp R (2007). Lifestyle-related factors and environmental agents causing cancer: an overview. Biomed Pharmacother.

[7] Coccia M (2013). The effect of country wealth on incidence of breast cancer. Breast Cancer Res Treat.

[8] Coccia M (2020). Factors determining the diffusion of COVID-19 and suggested strategy to prevent future accelerated viral infectivity similar to COVID. Sci Total Environ.

[9] Núñez-Delgado A, Bontempi E, Coccia M (2021). SARS-CoV-2 and other pathogenic microorganisms in the environment. Environ Res.

[10] Chang LW, Magos L, Suzuki T (1996). Toxicology of Metals.

[11] Prabhakaran KP, Cottenie A (1971). Parent material - soil relationship in trace elements - a quantitative estimation. Geoderma.

[12] han S, Cao Q, Zheng YM (2008). Health risks of heavy metals in contaminated soils and food crops irrigated with wastewater in Beijing, China. Environ Poll.

[13] Wuana RA, Okieimen FE (2011). Heavy metals in contaminated soils: a review of sources, chemistry, risks and best available strategies for remediation. International Scholarly Research Network ISRN Ecology.

[14] Li G, Sun GX, Ren Y (2018). Urban soil and human health: a review. Eur J Soil Sci.

[15] Nieder R, Benbi DK, Reichl FX (2018). Soil Components and Human Health.

[16] Rinklebe J, Antoniadis V, Shaheen SM (2019). Health risk assessmant of potentially oxic elements in soils along the Elbe River, Germany. Environ Int.

[17] Pohl WL (2020). Economic Geology. Principles and Practice.

[18] GWRTAC (1997). Remediation of metals-contaminated soils and groundwater, Tech Rep TE-97-01, GWRTAC, Pittsburgh, Pa, USA, 1997, GWRTAC-E Series.

[19] Aslanidis PSC, Golia EE (2022). Urban Sustainability at Risk Due to Soil Pollution by Heavy Metals—Case Study: Volos, Greece. Land.

[20] Golia EE, Papadimou SG, Cavalaris C (2021). Level of Contamination Assessment of Potentially Toxic Elements in the Urban Soils of Volos City (Central Greece). Sustainability.

[21] Serrani D, Ajmone-Marsan F, Corti G (2022). Heavy metal load and effects on biochemical properties in urban soils of a medium-sized city, Ancona, Italy. Environ Geochem Health.

[22] Ndoli A, Naramabuye F, Diogo RV (2013). Greenhouse experiments on soybean (Glycine max) growth on Technosol substrates from tantalum mining in Rwanda. Int J Agric Sci.

[23] Nieder R, Weber TKD, Paulmann I (2014). The geochemical signature of rare-metal pegmatites in the Central Africa Region: Soils, plants, water and stream sediments in the Gatumba tin-tantalum mining district, Rwanda. J Geochem Explor.

[24] Reetsch A, Naramabuye F, Pohl W (2008). Properties and quality of soils in the open-cast mining district of Gatumba, Rwanda. Etudes Rwandaises.

[25] Rossiter, DG (2007). Classification of Urban and Industrial Soils in the World Reference Base for Soil Resources. J Soils Sediments.

[26] Guo G, Li K, Lei M (2022). Accumulation, environmental risk characteristics and associated driving mechanisms of potential toxicity elements in roadside soils across China. Sci Total Environ.

[27] Antoniadis V, Levizou E, Shaheen SM (2017). Trace elements in the soil-plant interface: phytoavailability, translocation, and phytoremediation – a review. Earth Sci Rev.

[28] US EPA (Environmental Protection Agency) (2011). Reducing mercury pollution from gold mining.

[29] Pelfrêne A, Waterlot C, Mazzuca M (2012). Bioaccessibility of trace elements as affected by soil parameters in smelter-contaminated agricultural soils: A statistical modeling approach. Environ Pollut.

[30] Bolan N, Kunhikrishnan A, Thangarajan R (2014). Remediation of heavy metal(loid) contaminated soils – to mobilize or not to mobilize?. J Hazar Mater.

[31] Tong S, von Schirnding YE, Prapamontol T (2000). Environmental lead exposure: a public health problem of global dimensions. Bulletin of the World Health Organization.

[32] Järup L, Hellström L, Alfvén T (2000). Low level exposure to cadmium and early kidney damage: the OSCAR study. Occup Environ Med.

[33] Thomas LDK, Hodgson S, Nieuwenhuijsen M (2009). Early kidney damage in a population exposed to cadmium and other heavy metals. Environ Health Perspect.

[34] Putila JJ, Guo NL (2011). Association of arsenic exposure with lung cancer incidence rates in the United States. PLOS ONE.

[35] WHO (World Health Organization) (2016). News Release, Geneva.

[36] Liu P, Zhang Y, Feng N (2020). Potentially toxic element (PTE) levels in maize, soil, and irrigation water and health risks through maize consumption in northern Ningxia, China. BMC Public Health.

[37] Mazumder D (2008). Chronic arsenic toxicity and human health. Indian J Med Res.

[38] Bires J, Dianovsky J, Bartko P (1995). Effects on enzymes and the genetic apparatus of sheep after administration of samples from industrial emissions. Biol Met.

[39] Bolan S, Kunhikrishnan A, Seshadri B (2017). Sources, distribution, bioavailability, toxicity, and risk assessment of heavy metal(loid)s in complementary medicines. Environ Int.

[40] Rajkumar V, Gupta V, StatPearl (2022). Heavy Metal Toxicity.

[41] ATSDR (Agency for Toxic Substances and Disease Registry) (2000). Toxicological Profile for Arsenic.

[42] Tchounwou PB, Yedjou CG, Patlolla AK (2012). Heavy metal toxicity and the environment. Exp Suppl.

[43] WHO (World Health Organization) (2001). Arsenic and Arsenic Compounds. Environmental Health Criteria 224.

[44] Ravenscroft P, Brammer H, Richards K (2009). Arsenic Pollution: A Global Synthesis.

[45] Shakoor MB, Niazi NK, Bibi I (2019). Exploring the arsenic removal potential of various biosorbents from water. Environ Int.

[46] Shresther RR, Upadhyay NP, Pradhan R (2003). Groundwater arsenic contamination, its health impact and mitigation program in Nepal. J Environ Sci Health.

[47] Berg M, Trans HC, Nguyeu TC (2001). Arsenic contamination of groundwater and drinking water in Vietnam: a human health threat. Environ Sci Technol.

[48] Morton WE, Dunnette DA, Nriagu JO (1994). Health effects of environmental arsenic. Arsenic in the Environment Part II: Human Health and Ecosystem Effects.

[49] Zhao KL, Liu XM, Xu JM (2010). Heavy metal contaminations in a soil–rice system: Identification of spatial dependence in relation to soil properties of paddy fields. J Hazard Mater.

[50] Williams PN, Islam MR, Adomako EE (2006). Increase in rice grain arsenic for regions of Bangladesh irrigating paddies with elevated arsenic in ground waters. Environ Sci Technol.

[51] Tchounwou PB, Centeno JA, Patlolla AK (2004). Arsenic toxicity, mutagenesis and carcinogenesis - a health risk assessment and management approach. Mol Cell Biochem.

[52] Concha G, Vogler G, Nermell B (1998). Low-level arsenic excretion inbreast milk of native Andean women exposed to high levels of arsenic in the drinking water. Int Arch Occup Environ Health.

[53] Dakeishi M, Murata K, Grandjean P (2006). Long-term consequences of arsenic poisoning during infancy due to contaminated milk powder. Environ Health.

[54] Järup L, Åkesson A (2009). Current status of cadmium as an environmental healthproblem. Toxicol Appl Pharm.

[55] Inaba T, Kobayashi E, Suwazono Y (2005). Estimation of cumulative cadmium intake causing Itai-itai disease. Toxicol Lett.

[56] Skinner HCW (2007). The earth, source of health and hazards: An introduction to Medical Geology. Annu Rev Earth Planet Sci.

[57] Nakagawa H, Tabata M, Moikawa Y (1990). High mortality and shortened life-span in patients with itai-itai disease and subjects with suspected disease. Arch Environ Health.

[58] Horiguchi H, Oguma E, Sasaki S (2004). Comprehensive study of the effects of age, iron deficiency, diabetes mellitus and cadmium burden on dietary cadmium absorption in cadmium-exposed female Japanese farmers. Toxicol Appl Pharmacol.

[59] Diamond GL, Thayer WC, Choudhury H (2003). Pharmacokinetic/pharmacodynamics (PK/PD) modeling of risks of kidney toxicity from exposure to cadmium: estimates of dietary risks in the U.S. population. J Toxicol Environ Health.

[60] Dayan AD, Paine AJ (2001). Mechanisms of chromium toxicity, carcinogenicity and allergenicity: Review of the literature from 1985 to 2000. Hum Exp Toxicol.

[61] Coetzee JJ, Bansal N, Evans M N (2020). Chromium in environment, its toxic effect from chromite-mining and ferrochrome industries, and its possible bioremediation. Expos Health.

[62] Holmes AL, Wise SS, Wise JP (2008). Carcinogenicity of hexavalent chromium. Indian J Med Res.

[63] IARC (International Agency for Research on Cancer) (1990). Chromium, Nickel and Welding. IARC Monographs on the Evaluation of Carcinogenic Risks to Humans.

[64] Zhang JD, Li XL (1987). Chromium pollution of soil and water in Jinzhou. Zhonghua Yu Fang Yi Xue Za Zhi.

[65] Barregard L, Sallsten G, Conradi N (1999). Tissue levels of mercury determined in a deceased worker after occupational exposure. Int Arch Occup Environ Health.

[66] Ralston NVC, Raymond LJ (2018). Mercury's neurotoxicity is characterized by its disruption of selenium biochemistry. Biochim Biophys Acta Gen Subj.

[67] Sall ML, Diaw AKD, Gningue-Sall D (2020). Toxic heavy metals: impact on the environment and human health, and treatment with conducting organic polymers, a review. Environ Sci Pollut Res.

[68] Ekino S, Susa M, Ninomiya T (2007). Minamata disease revisited: An update on the acute and chronic manifestations of methyl mercury poisoning. J Neurol Sci.

[69] Eto K (2000). Minamata disease. Neuropathol.

[70] Babut M, Sekyi R (2003). Improving the environmental management of small-scale gold mining in Ghana: a case study of Dumasi. J Cleaner Prod.

[71] Taylor H, Appleton JD, Lister R (2004). Environmental assessment of mercury contamination from the Rwamagasa artisanal gold mining centre, Geita District, Tanzania. Sci Total Environ.

[72] WHO (World Health Organization) (2018). Lead poisoning and health.

[73] O'Connor D, Hou D, Ye J (2018). Lead-based paint remains a major public health concern: a critical review of global production, trade, use, exposure, health risk, and implications. Environ Int.

[74] Järup L (2003). Hazards of heavy metal contamination. Brit Med Bull.

[75] Shifaw E (2018). Review of Heavy Metals Pollution in China in Agricultural and Urban Soils. J Health Pollut.

[76] Wang W, Xu X, Zhou Z (2022). A joint method to assess pollution status and source-specific human health risks of potential toxic elements in soils. Environ Monit Assess.

[77] Lei M, Li K, Guo G (2022). Source-specific health risks apportionment of soil potential toxicity elements combining multiple receptor models with Monte Carlo simulation. Sci Total Environ.

[78] Xue S, Korna R, Fan J (2023). Spatial distribution, environmental risks, and sources of potentially toxic elements in soils from a typical abandoned antimony smelting site. J Environ Sci.

[79] Mohammadpour A, Emadi Z, Keshtkar M (2022). Assessment of potentially toxic elements (PTEs) in fruits from Iranian market (Shiraz): A health risk assessment study. J Food Compost Anal.

[80] Vejvodová K, Ash C, Dajčl J (2022). Assessment of potential exposure to As, Cd, Pb and Zn in vegetable garden soils and vegetables in a mining region. Sci Rep.

[81] Natasha N, Shahid M, Murtaza B (2022). Accumulation pattern and risk assessment of potentially toxic elements in selected wastewater-irrigated soils and plants in Vehari, Pakistan. Environ Res.

[82] Ghane ET, Khanverdiluo S, Mehri F (2022). The concentration and health risk of potentially toxic elements (PTEs) in the breast milk of mothers: a systematic review and meta-analysis. J Trace Elem Med Biol.

[83] DeForest DK, Brix KV, Adams WJ (2007). Assessing metal bioaccumulation in aquatic environments: the inverse relationship between bioaccumulation factors, trophic transfer factors and exposure concentration. Aquat Toxicol.

[84] Wang S, Shi X (2001). Molecular mechanisms of metal toxicity and carcinogenesis. Mol Cell Biochem.

[85] Ercal N, Gurer-Orhan H, Aykin-Burns N (2001). Toxic metals and oxidative stress Part I: mechanisms involved in metal-induced oxidative damage. Curr Top Med Chem.

[86] Wilk A, Kalisinska E, Kosik-Bogacka DI (2017). Cadmium, lead and mercury concentrations in pathologically altered human kidneys. Environ Geochem Health.

[87] Ebrahimpour M, Mosavisefat M, Mohabatti R (2010). Acute toxicity bioassay of mercuric chloride: an alien fish from a river. Toxicol Environ Chem.

[88] Brown SE, Welton WC (2008). Heavy metal pollution.

[89] Iyengar V, Nair P (2000). Global outlook on nutrition and the environment meeting the challenges of the next millennium. Sci Tot Environ.

[90] Turkdogan MK, Kulicel F, Kara K (2003). Heavy metals in soils, vegetable and fruit in the endermic upper gastro intestinal cancer region of Turkey. Environ Toxicol Pharmacol.

[91] Hindmarsh JT, Abernethy CO, Peters GR, Sarkar B (2002). Environmental Aspects of Arsenic Toxicity. Heavy Metals in the Environment.

[92] Apostoli P, Catalani S (2011). Metal ions affecting reproduction and development. Met Ions Life Sci.

[93] Rattan S, Zhou C, Chiang C (2017). Exposure to endocrine disruptors during adulthood: Consequences for female fertility. J Endocrinol.

[94] Bradl HB (2005). Heavy metals in the environment: origin, interaction and remediation.

[95] Davydova S (2005). Heavy metals as toxicants in big cities. Microchem J.

[96] Fergusson JE (1990). The heavy elements: Chemistry, environmental impact and health effects.

[97] Pierzynsky GM, Sims JT, Vance GF (2005). Soils and environmental quality.

[98] Wang LK, Chen JP, Hung Y (2009). Heavy metals in the environment.

[99] Sharma S, Kaur I, Kaur Nagpal A (2021). Contamination of rice crop with potentially toxic elements and associated human health risks—a review. Environ Sci Pollut Res.

[100] Shankar S, Shanker U, Shikha (2014). Arsenic contamination of groundwater: a review of sources, prevalence, health risks, and strategies for mitigation. Sci World J.

[101] Reichl FX, Ritter L (2011). Illustrated Handbook of Toxicology.

[102] NRC (National Research Council) (2001). Arsenic in drinking water-2001 update.

[103] Lauwerys RR, Hoet P (2001). Industrial Chemical Exposure. Guidelines for Biological Monitoring.

[104] Hughes MF (2002). Arsenic toxicity and potential mechanisms of action. Toxicol Lett.

[105] Kumagai Y, Sumi D (2007). Arsenic: signal transduction, transcription factor, and biotransformation involved in cellular response and toxicity. Annu Rev Pharmacol Toxicol.

[106] Huang HW, Lee CH, Yu HS (2019). Arsenic-induced carcinogenesis and immune dysregulation. Int J Environ Res Public Health.

[107] Kapaj S, Peterson H, Liber K (2006). Human health effects from chronic arsenic poisoning – a review. J Environ Sci Health Part A.

[108] Garza-Lombó C, Pappa A, Panayiotidis MI (2019). Arsenic-induced neurotoxicity: a mechanistic appraisal. J Biol Inorg Chem.

[109] Mitra S, Chakraborty AJ, Tareq AM (2022). Impact of heavy metals on the environment and human health: Novel therapeutic insights to counter the toxicity. Science.

[110] Kim YJ, Kim JM (2015). Arsenic toxicity in male reproduction and development. Dev Reprod.

[111] Salnikow K, Zhitkovich A (2008). Genetic and epigenetic mechanisms in metal carcinogenesis and cocarcinogenesis: Nickel, arsenic, and chromium. Chem Res Toxicol.

[112] Milton A, Hussain S, Akter S (2017). A review of the effects of chronic arsenic exposure on adverse pregnancy outcomes. Int J Environ Res Public Health.

[113] Roy J, Chatterjee D, Das N (2018). Substantial evidences indicate that inorganic arsenic is a genotoxic carcinogen: A review. Toxicol Res.

[114] Pierce BL, Kibriya MG, Tong L (2012). Genome-wide association study identifies chromosome 10q24.32 variants associated with arsenic metabolism and toxicity phenotypes in Bangladesh. PLoS Genet.

[115] Yin Y, Meng F, Sui C (2019). Arsenic enhances cell death and DNA damage induced by ultraviolet B exposure in mouse epidermal cells through the production of reactive oxygen species. Clin Exp Dermatol.

[116] Schwartz GG, Il'yasova D, Ivanova A (2003). Urinary cadmium, impaired fasting glucose, and diabetes in the NHANES III. Diabetes Care.

[117] Messner B, Knoflach M, Seubert A (2009). Cadmium is a novel and independent risk factor for early atherosclerosis mechanisms and in vivo relevance. Arterioscler Thromb Vasc Biol.

[118] Navas-Acien A, Selvin E, Sharrett AR (2004). Lead, cadmium, smoking, and increased risk of peripheral arterial disease. Circulation.

[119] Hellström L, Elinder CG, Dahlberg B (2001). Cadmium exposure and end-stage renal disease. Am J Kidney Dis.

[120] Everett CJ, Frithsen IL (2008). Association of urinary cadmium and myocardial infarction. Environ Res.

[121] Tellez-Plaza M, Navas-Acien A, Crainiceanu CM (2008). Cadmium exposure and hypertension in the 1999–2004 National Health and Nutrition Examination Survey (NHANES). Environ Health Perspect.

[122] Peters JL, Perlstein TS, Perry MJ (2010). Cadmium exposure in association with history of stroke and heart failure. Environ Res.

[123] Tellez-Plaza M, Guallar E, Howard BV (2013). Cadmium exposure and incident cardiovascular disease. Epidemiology.

[124] Branca JJV, Morucci G, Pacini A (2018). Cadmium-induced neurotoxicity: Still much to do. Neural Regen Res.

[125] Marchetti C (2014). Interaction of metal ions with neurotransmitter receptors and potential role in neurodiseases. Biometals.

[126] Wang BO, Du Y (2013). Cadmium and its neurotoxic effects. Oxid Med Cell Longevity.

[127] Nordberg GF, Nordberg M, Clarkson TW, Friberg L, Nordberg GF, Sager PR (2001). Biological monitoring of cadmium. Biological monitoring of toxic metals.

[128] Roels HA, Hoet P, Lison D (1999). Usefulness of biomarkers of exposure to inorganic mercury, lead, or cadmium in controlling occupational and environmental risks of nephrotoxicity. Ren Fail.

[129] Alfven T, Jarup L, Elinder CG (2002). Cadmium and lead in blood in relation to low bone mineral density and tubular proteinuria. Environ Health Perspect.

[130] Olsson IM, Bensryd I, Lundh T (2002). Cadmium in blood and urine – impact of sex, age, dietary intake, iron status, and former smoking – association of renal effects. Environ Health Perspect.

[131] Zalups RK (2000). Evidence for basolateral uptake of cadmium in the kidneys of rats. Toxicol Appl Pharmacol.

[132] Hyder O, Chung M, Cosgrove D (2013). Cadmium exposure and liver disease among US adults. J Gastrointest Surg.

[133] Jin T, Nordberg G, Ye T (2004). Osteoporosis and renal dysfunction in a general population exposed to cadmium in China. Environ Res.

[134] Fernandez MA, Sanz P, Palomar M (1996). Fatal chemical pneumonitis due to cadmium fumes. Occup Med.

[135] Davidson AG, Fayers PM, Newman Taylor AJ (1988). Cadmium fume inhalation and emphysema. Lancet.

[136] Mascagni P, Consonni D, Bregante G (2003). Olfactory function in workers exposed to moderate airborne cadmium levels. Neurotoxicol.

[137] Nishijo M, Nakagawa H, Honda R (2002). Effects of maternal exposure to cadmium on pregnancy outcome and breast milk. Occup Environ Med.

[138] Othumpamgat S, Kashon M, Joseph P (2005). Eukaryotic translation initiation factor 4E is a cellular target for toxicity and death due to exposure to cadmium chloride. J Biol Chem.

[139] WHO (World Health Organisation) (1990). Chromium. Environmental Health Criteria 61.

[140] Aaseth J, Alexander J, Norseth T (1982). Uptake of ^51^Cr chromate by human erythrocytes - a role of glutathione. Acta Pharmacol Toxicol.

[141] Mertz W (1969). Chromium occurrence and function in biological systems. Physiol Rev.

[142] Sharma BK, Singhal PC, Chugh KS (1978). Intravascular haemolysis and acute renal failure following potassium dichromate poisoning. Postgrad Med J.

[143] Glaser U, Hochrainer D, Kloppel H (1985). Low level chromium (VI) inhalation effects on alveolar macrophages and immune functions in Wistar rats. Arch Toxicol.

[144] Thompson CM, Fedorov Y, Brown DD (2012). Assessment of Cr(VI)-induced cytotoxicity and genotoxicity using high content analysis. PLoS ONE.

[145] Fang Z, Zhao M, Zhen H (2014). Genotoxicity of tri- and hexavalent chromium compounds in vivo and their modes of action on DNA damage in vitro. PLoS ONE.

[146] Sánchez-Sicilia L, Seto DS, Nakamoto S (1963). Acute mercury intoxication treated by hemodialysis. Ann Intern Med.

[147] Hong YS, Kim YM, Lee KE (2012). Methylmercury Exposure and Health Effects. J Prev Med Public Health.

[148] Cernichiari E, Brewer R, Myers GJ (1995). Monitoring methylmercury during pregnancy: maternal hari predicts fetal brain exposure. Neurotoxicol.

[149] Barregard L, Sallsten G, Schutz A (1992). Kinetics of mercury in blood and urine after brief occupational exposure. Arch Environ Health.

[150] Rahola T, Hattula T, Korolainen A (1973). Elimination of free and protein-bound ionic mercury (^203^Hg^2+^) in man. Ann Clin Res.

[151] NRC (National Research Council) (2000). Toxicological effects of methylmercury.

[152] Oskarsson A, Schultz A, Skerfving S (1996). Total and inorganic mercury in breast milk and blood in relation to fish consumption and amalgam fillings in lactating women. Arch Environ Health.

[153] Horowitz Y, Greenberg D, Ling G (2002). Acrodynia: a case report of two siblings. Arch Dis Child.

[154] Boyd AS, Seger D, Vannucci S (2000). Mercury exposure and cutaneous disease. J Am Acad Dermatol.

[155] Smith RG, Vorwald AJ, Pantil LS (1970). Effects of exposure to mercury in the manufacture of chlorine. Am Ind Hyg Assoc J.

[156] Smith PJ, Langolf GD, Goldberg J (1983). Effects of occupational exposure to elemental mercury on short term memory. Br J Ind Med.

[157] Tan SW, Meiller JC, Mahaffey KR (2009). The endocrine effects of mercury in humans and wildlife. Crit Rev Toxicol.

[158] Vupputuri S, Longnecker MP, Daniels JL (2005). Blood mercury level and blood pressure accrocc US women: results from the National Health and Nutrition Examination Survey 1999-2000. Environ Res.

[159] Yoshizawa K, Rimm EB, Morris JS (2002). Mercury and the risk of coronary heart disease in men. N Engl J Med.

[160] Salonen JT, Malin R, Tuomainen TP (1999). Polymorphism in high density lipoprotein paraoxonase gene and risk of acute myocardial infarction in men: Prospective nested case-control study. Br Med J.

[161] Kulka M (2016). A review of paraoxonase 1 properties and diagnostic applications. Pol J Vet Sci.

[162] Zefferino R, Piccoli C, Ricciard N (2017). Possible mechanisms of mercury toxicity and cancer promotion: involvement of gap junction intercellular communications and inflammatory cytokines. Oxid Med Cell Longev.

[163] Smith DR, Osterlod JD, Flegal AR (1996). Use of endogenous, stable lead isotopes to determine release of lead from the skeleton. Environ Health Perspect.

[164] Ettinger AS, Téllez-Rojo MM, Amarasiriwardena C (2004). Effect of Breast Milk Lead on Infant Blood Lead Levels at 1 Month of Age. Environ Health Perspect.

[165] Leggett RW (1993). Age-specific kinetic model of lead metal in humans. Environ Health Perspect.

[166] ATSDR (Agency for Toxic Substance and Disease Registry) (2007). Toxicological profile for lead.

[167] Muntner P, Vupputyuri S, Coresh J (2003). Blood lead and chronic kidney disease in the general United States population: results from NHANES III. Kidney Int.

[168] Hegazy AMS, Fouad UA (2014). Evaluation of lead hepatotoxicity; histological, histochemical and ultrastructural study. Forensic Med Anat Res.

[169] Hsiao CL, Wu KH, Wan KS (2011). Effects of environmental lead exposure on T-helper cell-specific cytokines in children. J Immunotoxicol.

[170] Silbergeld EK, Waalkes M, Rice JM (2000). Lead as a carcinogen: Experimental evidence and mechanisms of action. Am J Ind Med.

[171] Rousseau MC, Parent ME, Nadon L (2007). Occupational exposure to lead compounds and risk of cancer among men: A population-based case-control study. Am J Epidemiol.

[172] Pueschel SM, Linakis JG, Anderson AC (1996). Lead poisoning in childhood.

[173] Ling C, Ching Y, Hung-Chang L (2006). Effect of mother's consumption of traditional Chinese herbs on estimated infant daily intake of lead from breast milk. Sci Tot Environ.

[174] Carton JA (1988). Saturnismo. Med Clin.

[175] Larkin M (1997). Lead in mothers' milk could lead to dental caries in children. Sci Med.

[176] Canfield RL, Henderson CR, Cory-Slechta DA (2003). Intellectual impairment in children with blood lead concentrations below 10 µg dL^−1^. N Engl J Med.

[177] Schwartz BS, Lee BK, Lee GS (2001). Associations of blood lead, dimercaptosuccinic acid-chelatable lead, and tibia lead with neurobehavioral test scores in South Korean lead workers. Am J Epidemiol.

[178] Vaziri ND (2008). Mechanisms of lead-induced hypertension and cardiovascular disease. Am J Physiol.

[179] Bellinger D (2005). Teratogen update: lead and pregnancy. Birth Defects Research.

[180] Borja-Aburto VH, Hertz-Picciotto I, Rojas LM (1999). Blood lead levels measured prospectively and risk of spontaneous abortion. Am J Epidemiol.

[181] Telisman S, Cvitkovic P, Jurasovic J (2000). Semen quality and reproductive endocrine function in relation to biomarkers of lead, cadmium, zinc and copper in men. Environ Health Perspect.

[182] Kim JJ, Kim YS, Kumar V (2019). Heavy metal toxicity: An update of chelating therapeutic strategies. J Trace Elem Med Biol.

[183] Sears ME (2013). Chelation: harnessing and enhancing heavy metal detoxification - a review. Sci World J.

[184] Amadi CN, Offor SJ, Frazzoli C (2019). Natural antidotes and management of metal toxicity. Environ Sci Pollut Res.

[185] Rafati Rahimzadeh M, Rafati Rahimzadeh M, Kazemi S (2017). Cadmium toxicity and treatment: An update. Caspian J Intern Med.

[186] Singh KP, Bhattacharya S, Sharma P (2014). Assessment of heavy metal contents of some Indian medicinal plants. Am Eurasian J Agric Environ Sci.

[187] Bhattacharya S (2018). Medicinal plants and natural products can play a significant role in mitigation of mercury toxicity. Interdiscip Toxicol.

[188] Bhattacharya S, Haldar PK (2012). *Trichosanthes dioica* root possesses stimulant laxative activity in mice. Nat Prod Res.

[189] Bhattacharya S, Haldar PK (2012). Protective role of the triterpenoid-enriched extract of Trichosanthes dioica root against experimentally induced pain and inflammation in rodents. Nat Prod Res.

